# Gut bacterial and fungal dysbiosis in tuberculosis patients

**DOI:** 10.1186/s12866-024-03275-8

**Published:** 2024-04-25

**Authors:** MeiQing Han, Xia Wang, JiaMin Zhang, Lin Su, Hafiz Muhammad Ishaq, Duan Li, JunWei Cui, HuaJie Zhao, Fan Yang

**Affiliations:** 1 Department Four of Tuberculosis Medicine, The First Affiliated Hospital, Xinxiang Medical University, Xinxiang, China; 2 Department of Pathogenic Biology, School of Basic Medical Science, Xinxiang Medical University, Xinxiang, China; 3 Department of Pathobiology, Faculty of Veterinary and Animal Sciences, Muhammad Nawaz Shareef University of Agriculture, Multan, Pakistan

**Keywords:** Tuberculosis, Gut microbiota, Gut mycobiota, Dysbiosis, IFN-γ, IL-17

## Abstract

**Background:**

Recent studies have more focused on gut microbial alteration in tuberculosis (TB) patients. However, no detailed study on gut fungi modification has been reported till now. So, current research explores the characteristics of gut microbiota (bacteria)- and mycobiota (fungi)-dysbiosis in TB patients and also assesses the correlation between the gut microbiome and serum cytokines. It may help to screen the potential diagnostic biomarker for TB.

**Results:**

The results show that the alpha diversity of the gut microbiome (including bacteria and fungi) decreased and altered the gut microbiome composition of TB patients. The bacterial genera *Bacteroides and Prevotella* were significantly increased, and *Blautia and Bifidobacterium* decreased in the TB patients group. The fungi genus *Saccharomyces* was increased while decreased levels of *Aspergillus* in TB patients. It indicates that gut microbial equilibrium between bacteria and fungi has been altered in TB patients. The fungal-to-bacterial species ratio was significantly decreased, and the bacterial-fungal trans-kingdom interactions have been reduced in TB patients. A set model including *Bacteroides, Blautia, Eubacterium_hallii_group, Apiotrichum, Penicillium*, and *Saccharomyces* may provide a better TB diagnostics option than using single bacterial or fungi sets. Also, gut microbial dysbiosis has a strong correlation with the alteration of IL-17 and IFN-γ.

**Conclusions:**

Our results demonstrate that TB patients exhibit the gut bacterial and fungal dysbiosis. In the clinics, some gut microbes may be considered as potential biomarkers for auxiliary TB diagnosis.

**Supplementary Information:**

The online version contains supplementary material available at 10.1186/s12866-024-03275-8.

## Background

Tuberculosis (TB) is a respiratory infectious disease caused by *Mycobacterium tuberculosis* (*M.tb*). TB is one of the leading sources of death from a single infectious source and is ranked the 13th dominant cause of death worldwide [1]. According to the World Health Organization (WHO) report, approximately 10.6 million people worldwide have suffered active TB, and 1.13 million deaths occur annually [2]. China is one of the most affected countries in the world with a high prevalence of TB. According to the China Health Statistics Yearbook (2018), China is the 3rd most affected country globally due to the high number of TB reported cases [3]. the emergence of multidrug-resistant, TB requires more effective interventions to prevent and for treatment of TB. Hence, it is the need of our to study and find the diagnosis and prevention of TB from various aspects.

Many factors influenced the occurrence and development of TB, including the virulence of *M.tb*, the host’s immunity, and environmental factors [4, 5]. Recently, researchers have focused on the gut microbiome as a crucial host-associated factor in the occurrence of many infectious diseases and TB [6–8]. The human body harbours many microbial communities, including bacteria, fungi, and viruses, which perform different important functions, i.e. nutritional, metabolic, and immunological processes [9–11]. It has recently been reported that the gut microbiome may influence the intestinal tract and other organs like the lungs [12]. According to the “gut-lung axis” theory, it has been well recognized that gut microbiota plays a vital role in pulmonary infectious disease [13, 14].

Recently, many studies have been reported on gut microbial alteration in TB patients and with the treatment of anti-tuberculous drugs. Most of the reported literature indicated that the alpha diversity of the intestinal microbiota showed decreased levels in TB patients. However, some research also exhibited the contrary results [15–17]. Wang et al. reported that the abundance of *Bacteroides* and *Lachnoclostridium* was higher, while the abundance of *Blautia* and *Bifidobacterium* was lower in TB patients compared with the healthy subjects [16]. However, Khaliq et al. found that the relative abundance of *Bifidobacterium* was raised in the TB patients [18]. Another research on the gut microbiota of children TB patients showed increased levels of pro-inflammatory bacteria like *Prevotella*, while *Ruminococcaceae*, *Bifidobacteriaceae*, and *Prausnitzii* showed a decreased trend [19]. Although there is much literature available focusing on the gut microbiota of TB patients, the results of these reported studies were not consistent in TB patients. In addition, most of the research mainly emphasized the gut bacterial alteration of TB patients. However, literature on gut fungi alteration is scarce in TB patients. Hence, it is important to identify gut bacterial and fungi dysbiosis characteristics in TB patients, which may provide clear guidelines for the understanding of TB development.

In the present study, we enrolled 33 tuberculosis patients and 20 healthy volunteers to investigate the alteration of gut microbiota (bacteria) and mycobiota (fungi) by using 16 S rRNA and ITS gene sequencing and try to screen the potential diagnostic biomarker of TB. Furthermore, we also investigated the levels of IFN-γ and IL-17 in the peripheral blood of enrolled subjects and analyzed the correlation between gut microbiome dysbiosis and immune cytokines.

## Materials and methods

### Research protocols and ethical statements

33 active TB patients and 20 healthy control subjects (HC) were enrolled in the current research. All TB patients were taken from the first affiliated hospital of Xinxiang Medical University from October 2022 to February 2023. The healthy control participants were recruited from the population of physical examination in the first affiliated hospital of Xinxiang Medical University from January to February 2023. All subjects had no history of smoking and alcohol consumption, and all participants did not have HIV infection, diabetes, lung diseases, and gastrointestinal diseases. None of the enrolled participants had received probiotics, prebiotics, or antibiotics one month before hospital admission and evaluation. Basic demographic and clinical data were collected at the time of initial hospital evaluation for all subjects (Table S1). The study protocol received ethics approval from the Human Research Ethics Committee of the first affiliated hospital of Xinxiang Medical University (EC-023-089). Written informed consent was taken from all research subjects before sample collection.

### Fecal sample collection and DNA extraction

Fresh stool samples (morning samples) from the volunteers were collected using a sterile container and stored at −80°C until DNA extraction. The total genomic DNA from fecal samples was extracted by using the QIAamp DNA Stool Mini kit (QIAGEN, Inc., Netherlands) according to the manufacturer’s instructions. The quality and concentration of DNA were determined using NanoDrop® ND-2000 spectrophotometer (Thermo Scientific Inc, USA) and electrophored in.1.0% agarose gel. Finally, all DNA samples were stored at −20°C until further processing.

### 16S rRNA and ITS gene high-throughput sequencing

For gut bacterial analysis, the universal primer (338F, 5’-ACTCCTACGGGAGGCAGCAG-3; 806R, 5’-GGACTACNNGGGTATCTAAT-3’) was used to amplify the V3-V4 hypervariable regions of the 16 S rRNA genes through PCR. For gut fungi, the internal transcribed spacer region 2 (ITS2) was amplified with the primer of ITS3F/ITS4R (ITS3F, 5’-GCATCGATGAAGAACGCAGC-3’; ITS4R, 5’-TCCTCCGCTTATTGATATGC-3’). The PCR products were purified with the AxyPrep DNA Gel Extraction Kit (Axygen Biosciences, Union City, CA, USA) and quantified using Quantus™ Fluorometer (Promega, USA). The amplicons were sequenced on the MiSeq PE300 platform (Illumina, San Diego, CA, USA) according to the standard protocols provided by Majorbio Bio-Pharm Technology Co., Ltd. (Shanghai, China).

### Serum cytokine measurement

The blood samples of all subjects were collected via venipuncture, and the serum was separated by centrifugation at 1000×g for 20 min. Then, ELISA was used to measure the serum cytokine including IFN-γ and IL-17, according to the manufacturer’s instructions. Commercial ELISA kits were purchased from Shanghai Enzyme-linked Biotechnology Co., Ltd (Shanghai, China). IFN-γ (catalog number YJ064303), IL-17 (catalog number YJ027426).

### Bioinformatics and statistical analysis

All bioinformatic analysis of the gut microbiome was performed on the Majorbio Cloud online platform (https://cloud.majorbio.com). The alpha diversity like the Simpson and Chao 1 index was assessed to study gut microbial diversification. The beta-diversity indices (analyzed using PCoA) were calculated based on Unweighted uniFrac distances at the genus level. A permutational analysis of variance (PERMANOVA) was performed to assess the variation in the taxonomic composition of microbiota communities between the groups. LDA and LEfSe analyses were performed to compare biomarkers between the groups. Correlation analyses were performed using Spearman`s rho correlation test. The correlations among main gut bacterial genera, fungi genera, and cytokines were assessed by linear regression analysis. The trans-kingdom network figures were built by using the package igraph (version 1.2.6). The linear regression analysis was applied to plot the ROC curve in SPSS (version 22.0) software. Calculation of the area under the curve (AUC) and 95% confidence interval (CI) were used to predict biomarkers in order to distinguish TB from HC. The Welch’s t-test was computed to screen for differential metabolic pathways by STAMP software. Other statistical analyses were performed using GraphPad Prism software Version 8.0. Unpaired t-tests (for standard data) or Mann-Whitney U test (for uneven data) were performed when the two groups were compared.

## Results

### Demographic characteristics of the study participants

A total of 33 active TB patients and 20 age- and sex-matched healthy subjects were recruited in this study. All subjects belonged to the Han ethnic group, they had the same lifestyle, and they had no history of smoking and alcohol consumption. Table 1 shows the detailed characteristics of all the study participants. The demographics including nationality, age, sex, and body mass index (BMI) were comparable between the two groups (Table 1, Table S1). The sputum culture was positive for all enrolled TB patients, and 93.93% (31/33) of TB patients showed lung pathological symptoms by chest x-rays. 51.51% (17/33) of TB patients suffered from cough, 30.30% (10/33) patients with fever, and only 3 patients presented with hemoptysis. T-spot assay showed that 57.58% (19/33) of TB patients were positive and all healthy control subjects were negative (Table 1). All the detailed sociodemographic and medical characteristics of the participants are shown in Table S1.

**Table 1:** Demographic and clinical characteristics of the participants

Parameters		TB (*n* = 33)	HC (*n* = 20)	P-value
Nationality		Han	Han	
Gender	Male	20	10	
	Female	13	10	0.45
Age		40.91 ± 20.52	39.65 ± 10.6	0.9167
BMI		20.57 ± 4.14	21.17 ± 2.49	0.3094
Smoking		0	0	
Alcohol consumption		0	0	
Cough		17	0	–
Fever		10	0	–
Hemoptysis		3	0	–
Smear positive (%)		23	0	–
Sputum-culture positive (%)		33	0	–
T-spot positive (%)		19	0	–
Chest X-rays positive (%)		31	0	–

Data are expressed as the mean ± SD. BMI, body mass index, and Chest X-rays positive include infiltrate, cavitary lesions, and miliary pattern. The U Mann–Whitney test evaluated the significant difference between the two groups. TB: tuberculosis; HC: healthy control

### Overview of gut bacterial and fungal sequence composition in TB patients and health subjects

For gut bacterial sequencing, after quality control and chimera filtering, a total of 3,764,330 reads were obtained in two groups. On average 63,761 and 83,009 sequences per sample were obtained in TB patients and healthy subjects, respectively. Mann-Whitney U test showed significant differences in sequencing reads between the two groups (*p* < 0.0001) (Table 2). We identified an average of 207 Amplicon Sequence Variant (ASVs) per sample in TB patients and 153 ASVs in the healthy control group, respectively. The ASVs showed no significant differences between the two groups (Table 2). For gut fungi sequencing, a total of 3,980,553 reads were obtained by ITS sequencing. The average number of sequences in the healthy control group was 121,607 per sample, significantly higher than in the TB group. An average of 44 ASVs per sample were obtained from the TB group, with less than 110 ASVs in the HC group (Table 2). These results indicated that the sequence composition of the gut microbiome (including bacteria and fungi) was altered in TB patients.

**Table 2:** Bacterial 16S rRNA and fungal ITS DNA sequencing data summary

	Bacteria 16s rRNA		Fungi ITS	
	TB	HC	TB	HC
Total Reads	2,104,142	1,660,188	1,913,224	2,067,329
Min reads/sample	1,034	70,127	45,596	90,857
Max reads/sample	75,931	100,356	110,968	154,794
Mean reads/sample	63,761 ± 7,195	83,009 ± 8,380	68,329 ± 15,244	121,607 ± 27,058
P-value	< 0.0001		< 0.0001	
ASVs	207 ± 150	153 ± 42	44 ± 40	110 ± 60
P-value	0.76		0.001	

Data are expressed as the mean ± SD. Mann-Whitney U test was used to compare the differences between TB and HC groups. TB: tuberculosis; HC: healthy control

### The diversity and composition of gut bacteria were altered in TB patients

**Fig. 1:**
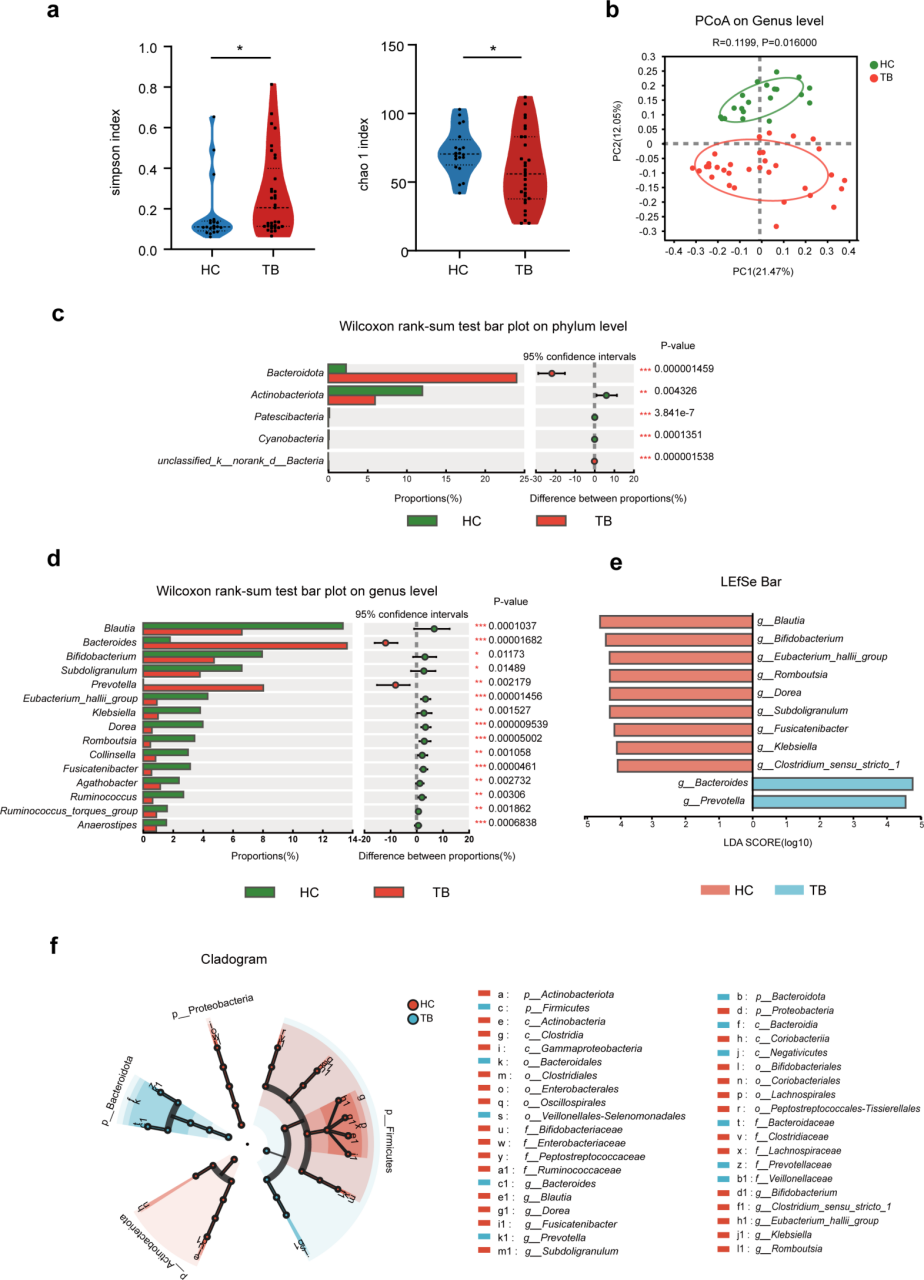
Comparison of TB patients’ intestinal bacterial diversity and composition versus healthy controls. (**a**) Simpson index and Chao 1 index represent the alpha diversity (Mann-Whitney U test). (**b**) The beta diversity was assessed using PCoA, which is based on Unweighted unifrac distance. ANOSIM was used to analyze the differences in the bacterial community between the TB group and the HC group. Comparisons of the relative abundances of intestinal bacteria between the TB and HC groups were conducted at the phylum (**c**) and genus (**d**) levels (Wilcoxon Rank Sum test). The LEfSe analysis (**e**) identified the differentially abundant taxa between the TB patients and HC (LDA > 4, *P* < 0.05). The taxa enriched in the HC were characterized by a positive LDA score (red), and the TB-enriched taxa were indicated with a negative LDA score (blue). The circular cladogram (**f**) is a taxonomic diagram showing the taxonomic hierarchy of the signified species in patients with TB and HC by LEfSe. TB: tuberculosis; HC: healthy control. *P < 0.05; **P < 0.01; ***P < 0.001

The Simpson and Chao 1 indexes were used to evaluate the alpha diversity of gut microbiota. The results showed that the alpha diversity significantly decreased in the TB group compared with the HC group (Fig. 1a). Based on the Unweighted unifrac distance, the principal coordinate analysis (PCoA) exhibited distinctly different clustering results of samples between the TB group and the HC group (Fig. 1b). It clearly indicates that microbial community composition has been altered in TB patients. We analyzed the gut microbial composition at different taxonomic levels to assess which bacteria are predominantly altered in TB patients. At the phylum level, the abundance of *Bacteroidetes* was notably increased while *Actinobacteria* and *Proteobacteria* were significantly decreased in TB patients compared with the healthy subjects (Fig. 1c, Table S2). At the genus level, the variation trend of dominant bacterial genera (an abundance > 0.1% in the two groups) is shown in Table S3. Among the dominant microbiota, the abundance of *Bacteroides, Prevotella*, and *Feacalibacterium* was higher, and the abundance of *Blautia*, *Bifidobacterium*, and *Eubacterium_hallii_group* were lowered in the TB group vs. HC group (Fig. 1d). To better understand the difference in microbiota between the two groups, we further performed a LEfSe analysis according to a linear discriminant analysis (LDA) score ≥ 4.0. The results of LEfSe analysis showed that 11 key genera were different between the two groups (Fig. 1e). The cladogram Figure (from phylum to genus level) shows that altered genera mainly belonged to *Bacteroidetes, Firmicutes*, and *Proteobacteria* (Fig. 1f). Overall, our data indicate that the gut bacterial diversity and composition have been altered in TB patients.

### Altered gut mycobiota profile in TB patients

**Fig. 2:**
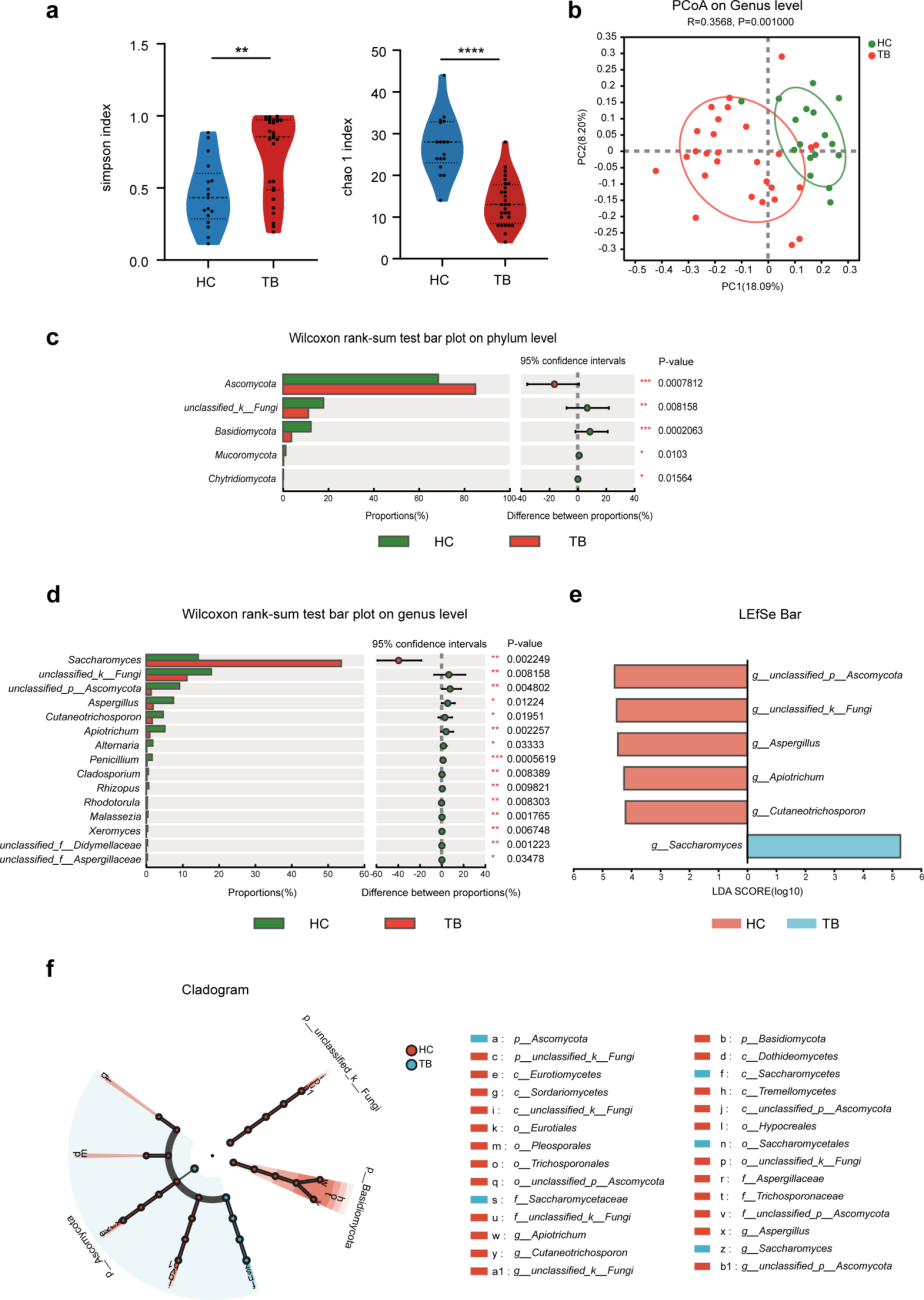
Comparison of TB patients’ gut fungal diversity and composition versus healthy controls. (**a**) Simpson index and Chao 1 index represent the alpha diversity (Unpaired t-tests). (**b**) The beta diversity was assessed using PCoA analysis based on Unweighted unifrac distance. ANOSIM was used to analyze the differences in the fungal community between the TB group and the HC group. Comparisons of the relative abundances of intestinal fungus between the TB and HC groups were conducted at the phylum (**c**) and genus (**d**) levels (Wilcoxon Rank Sum test). (**e**) The LEfSe analysis identified the differentially abundant taxa between the TB patients and HC (LDA > 4, *P* < 0.05). (**f**) The circular cladogram is a taxonomic diagram showing the taxonomic hierarchy of the signified species in patients with TB and HC by LEfSe. PCoA, principal coordinate analysis; LEfSe, linear discriminant analysis effect size; LDA, linear discriminant analysis; TB: tuberculosis; HC: healthy control. *P < 0.05; **P < 0.01; ***P < 0.001

We assessed the alteration of the mycobiome in TB patients using ITS2 sequencing. The results indicate that the TB group has lowered alpha diversity as compared to the HC group (Fig. 2a). Based on Unweighted unifrac distance, PCoA analysis showed that the TB patient samples were distinguished from HC samples (Fig. 2b). The results of ANOSIM were *R* = 0.3568 and *P* = 0.001, indicating that the gut mycobiota composition was significantly dissimilar between the two groups. Further analysis of the community structure of the mycobiota found that *Ascomycota* was the dominant phylum in the two groups. The relative abundance of *Ascomycota* was notably increased in the TB group vs. the HC group (84.89% vs. 68.46%, *p* < 0.001, Fig. 2c, Table S4). At the genus level, *Saccharomyces* and *Candida* were the most abundant genera in health subjects (14.28%, and 22.26%, respectively). The abundance of *Saccharomyces* was increased to 53.67% in the TB patients. *Aspergillus*, *Cutaneotrichosporon*, and *Apiotrichum* were substantially reduced in the TB group compared with the HC group (Table S5 and Fig. 2d). With the LEfSe analysis, *Saccharomyces* were significantly enriched and 5 fungal genera (including *Aspergillus, Cutaneotrichosporon*, and *Apiotrichum*) were diminished in the TB patients vs. healthy subjects (Fig. 2e). The cladogram Figure shows that all these altered genera belonged to *Ascomycota* and *Basidiomycota* (Fig. 2f). The results suggest that the diversity index of gut fungi shows a decreased trend while the composition of gut mycobiota is altered in TB patients.

### The bacterial-fungal trans-kingdom network alter in TB patients

**Fig. 3:**
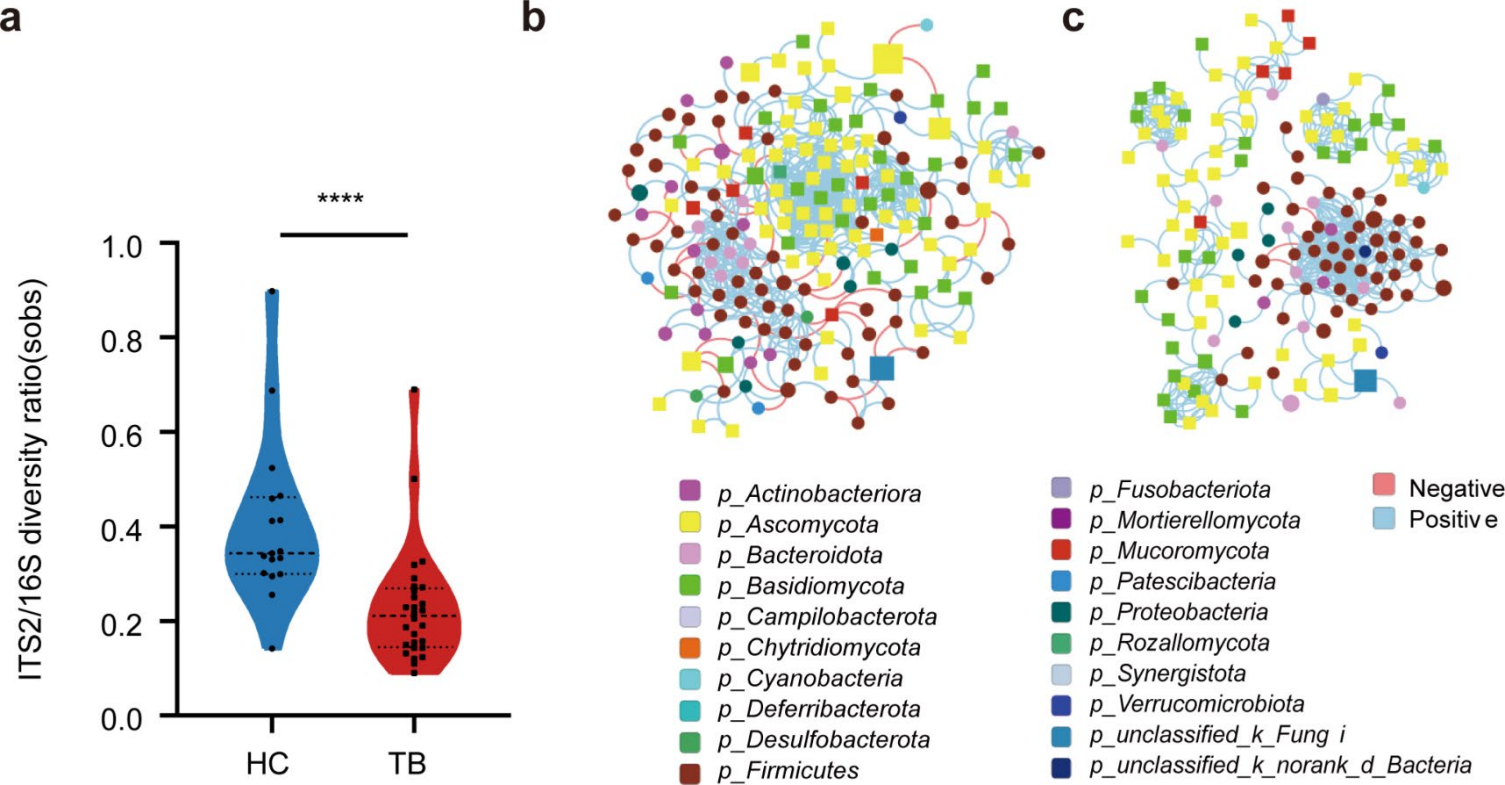
Fungal-bacterial equilibration analyses in two groups. (**a**) The ITS2/16S diversity ratio was calculated by the Sobs at the genus level (Mann-Whitney U test). Trans-kingdom abundance correlation networks of HC (**b**) and TB (**c**) groups at the genus level using the Spearman coefficient. Each node represents a genus, and the color of the nodes represents the phylum to which it belongs. Node’s shape represents the kingdom to which they belong (square is fungus and circle is bacteria). The size of the node represents the mean abundance of the genus. Edge represents the positive correlations (blue) and negative correlations (red). ****P < 0.0001

To assess the changes in the gut fungal and bacterial diversity balance in the TB patients, we analyzed the fungal-to-bacterial species ratio based on observed Sobs indexes of the ITS/16S. The results showed that the ITS/16S ratio of the TB group was significantly decreased as compared with the HC group (Fig. 3a), which suggested that the equilibrium between fungi and bacteria in the gut was altered in the TB patients. However, the trans-kingdom network analysis between bacteria and fungi was performed to assess the interaction at the genus level. The results showed that the trans-kingdom networks differed between the two groups (Fig. 3b-c). In the HC group, a total of 193 nodes were found at the genus level. The ratio of fungi and bacteria was 1.05 (99/94), shown in (Table 3). These bacteria and fungi were related to each other gathering in a cluster and forming a more complex network (Fig. 3b). However, the centralization and density of the trans-kingdom network in the TB group were dramatically decreased compared with the HC group (Fig. 3c). There were 153 nodes and 468 edges. The interaction ratio between bacteria and fungi, fungi and fungi was also remarkably reduced in this network (Table 3). Interestingly, the negative correlations between bacteria and fungi were rare in the TB group (Fig. 3c). All these results indicated that the bacterial-fungal trans-kingdom interactions were reduced, and the equilibrium of the entire ecosystem (microbiome) in the gut was altered in the TB patients.

**Table 3:** Parameters of the trans-kingdom abundance correlation networks

	HC	TB
Nodes (n) (fungi/bacteria)	193 (99/94)	153 (82/71)
Edges (n)	573	468
Relative connectedness	2.97	3.06
Bacteria-bacteria (%)	32.46	51.07
Bacteria-fungi (%)	12.57	10.04
Fungi-fungi (%)	54.97	38.89

The relative connectedness of the network was calculated by the ratio of edges (the number of significant interactions) and nodes (the number of genera). Bacteria-bacteria (%) of the network was computed by the ratio of edges (Bacteria-bacteria) and edges (the number of significant interactions). Bacteria-fungi (%) of the network was calculated by the ratio of edges (Bacteria-fungi) and edges (the number of significant interactions). Fungi-fungi (%) of the network was calculated by the ratio of edges (Fungi-fungi) and edges (the number of significant interactions). TB: tuberculosis; HC: healthy control

### Screening microbiota biomarkers for TB diagnosis and potential functional prediction based on gut microbiome

**Fig. 4:**
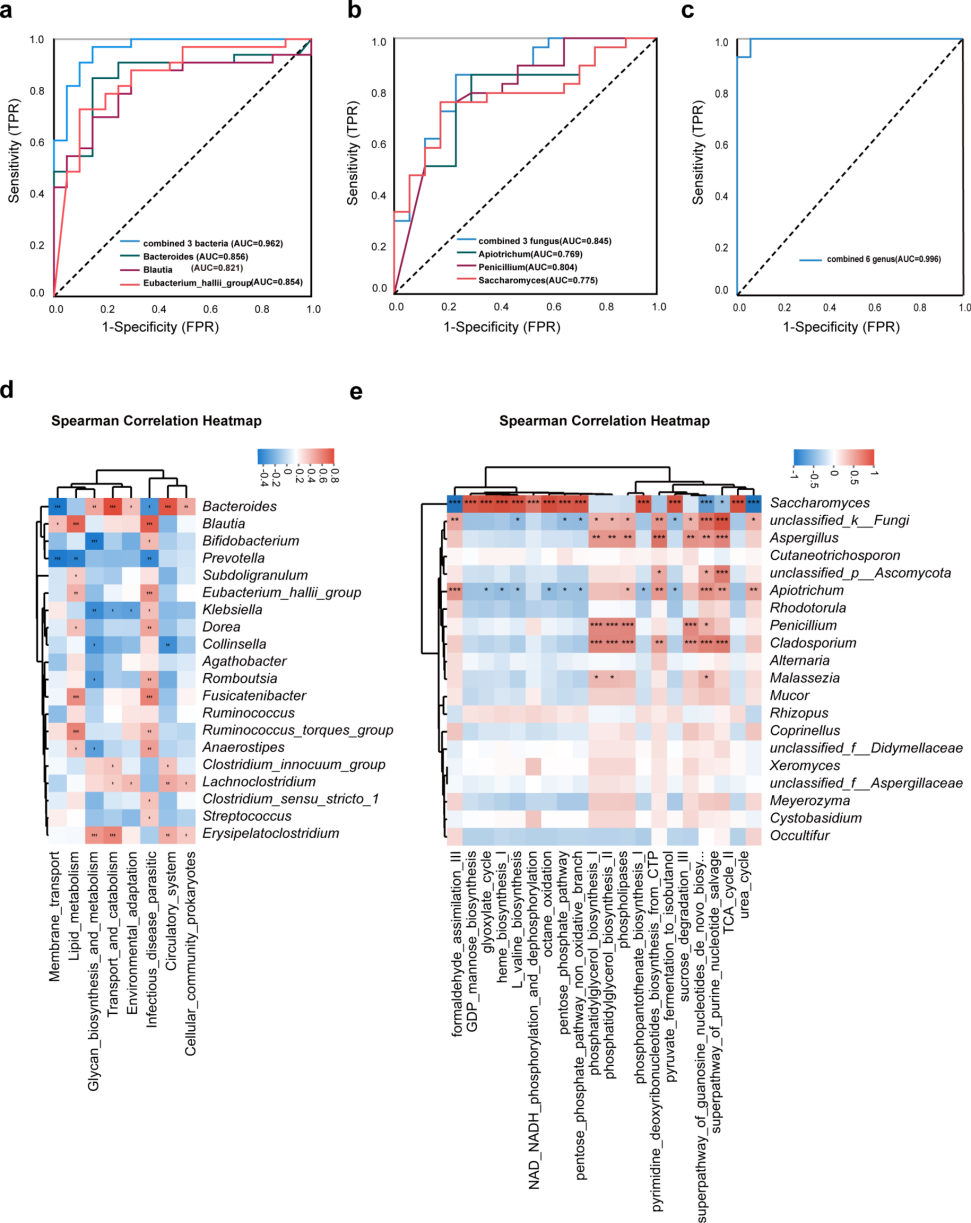
ROC analysis and functional prediction of gut microbiome. ROC analysis of (**a**) bacteria, (**b**) fungi, and (**c**) the combination of fungi and bacteria. Correlation between predicted differential pathways and the top 20 differential bacteria (**d**) and fungi (**e**). The depth of color in the heat map indicates the strength of the correlation: red indicates a positive correlation, and blue indicates a negative correlation. ROC: Receiver operating characteristic; AUC: area under the curve (0.5–1.0). *P < 0.05; **P < 0.01; ***P < 0.001; ****P < 0.0001

To explore the characteristics of gut microbiomes in TB patients as compared to healthy subjects. Firstly, we screened 75 differential bacterial genera between the TB and HC groups for ROC analysis to verify the diagnostic ability of these biomarkers. The results show three bacterial genera *Bacteroides* (AUC = 0.856, 95%CI: 0.749–0.963), *Blautia* (AUC = 0.821, 95%CI:0.706–0.936), and *Eubacterium_hallii_group* (AUC = 0.854, 95%CI: 0.746–0.962) have a potential capacity to distinguish TB patients from healthy human (Fig. 4a). Then, we selected 30 different fungal genera to perform ROC analysis. The results showed that *Apiotrichum* (AUC = 0.769, 95%CI:0.622–0.916), *Penicillium* (AUC = 0.804, 95%CI:0.666–0.941), and *Saccharomyces* (AUC = 0.775, 95%CI:0.638–0.912) have the strong diagnostic ability for TB patients (Fig. 4b). Interestingly, the combination of the above 6 bacterial and fungal genera has a better diagnostic capacity (AUC = 0.996, 95%CI:0.985–1.000) than using single bacterial or fungi sets (Fig. 4c).

Using PICRUSt2 (Phylogenetic Investigation of Communities by Reconstruction of Unobserved States), we predicted the potential enriched pathways of the microbiome based on the 16 S rRNA and ITS2 gene. A total of 45 level-II metabolic pathways were obtained in all samples of bacteria in function prediction. Compared with the HC group, there were 8 metabolic pathways altered in TB patients. Glycan biosynthesis and metabolism were significantly increased in the TB group (Figure S1). ITS2 functional predicating showed the 20 differential pathways between HC and TB groups. Metabolic functions involved in octane oxidation, glyoxylate cycle, NAD/NADH phosphorylation and dephosphorylation were increased in TB patients compared to the healthy control group (Figure S2).

Then, correlation analysis was performed between predicted metabolic pathways and the top 20 differential microbiota. The results showed that *Bacteroides* were positively correlated with the pathway of transport and catabolism, and Glycan biosynthesis and metabolism, but strongly negatively correlated with the membrane transport pathway. *Bifidobacterium* was negatively correlated with glycan biosynthesis and metabolism pathways (Fig. 4d). *Saccharomyces* was positively correlated with the Glyoxylate cycle, octane oxidation, and NAD/NADH phosphorylation pathways. However, it was negatively correlated with formaldehyde assimilation III and urea cycle pathway (Fig. 4e). These results suggest that gut microbial dysbiosis in TB patients induces the alteration of the gastrointestinal tract metabolism pathways.

### Correlations between the gut microbiota and peripheral cell cytokines

**Fig. 5:**
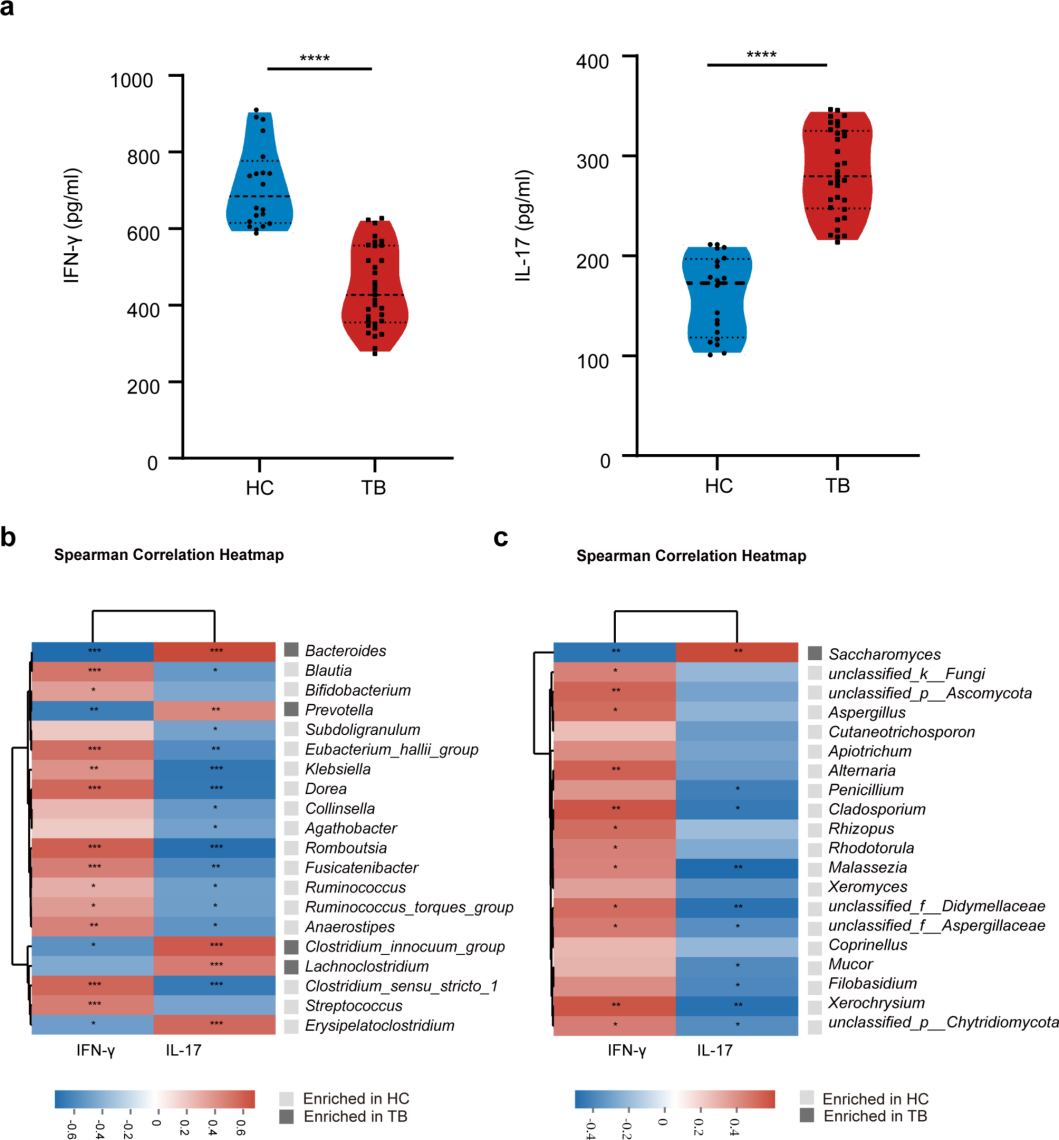
Correlation analysis between intestinal flora and serum cytokine. (**a**) Compared cytokine levels of IFN-γ and IL-17 in serum between two groups ( Mann-Whitney U test). (**b**) Correlation analysis was performed between the relative abundance of bacteria and the levels of IFN-γ and IL-17. (**c**) Spearman was used to evaluate the correlation between fungus and IFN-γ and IL-17. The depth of color in the heat map indicates the strength of the correlation: red indicates a positive correlation, while blue indicates a negative correlation. *P < 0.05; **P < 0.01; ***P < 0.001

We detect the levels of IFN-γ and IL-17 in the peripheral serum of TB patients and healthy subjects. The results showed that IFN-γ decreased and IL-17 increased in the TB group vs. HC group (Fig. 5a). To further investigate whether these alterations correlate with gut microbial alteration or not. we perform correlation analyses based on Pearson’s coefficients. The results show *Bacteroides*, *Prevotella*, *Clostridium_innocuum_group*, and *Lachnoclostridium* were enriched in the TB group, positively correlated with IL-17, and negatively correlated with IFN-γ (Fig. 5b). However, *Dorea*, *Romboutsia*, and *Klebsiella* showed contrary results to the above (Fig. 5b).

Similarly, the correlation between fungi and cell cytokines showed that *Saccharomyces* (enriched in the TB group) was negatively correlated with IFN-γ and positively correlated with IL-17. *Penicillium*, *Cladosporium*, and *Xerochrysium* showed the contrary results (Fig. 5c). All these results reaffirm the potential relationship between intestinal microbial dysbiosis and serum cytokines alteration in TB patients.

**Fig. 6:**
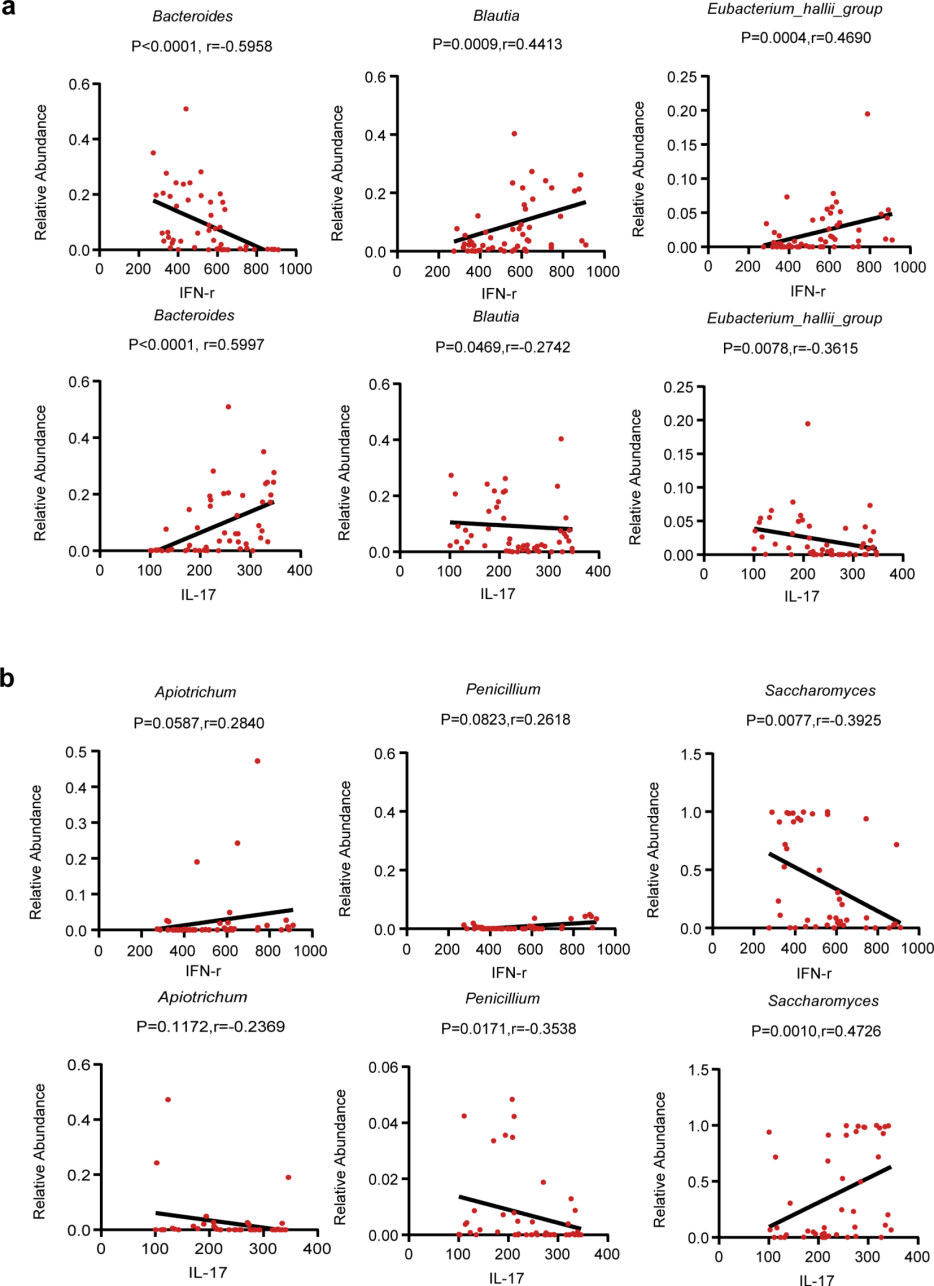
Linear Regression analysis between diagnostics biomarkers and serum cytokine. (**a**) Linear Regression between the relative abundance of *Bacteroides*, *Blautia*, and *Eubacterium_hallii_group* with IFN-γ and IL-17 expression. (**b**) Linear Regression between the relative abundance of *Apiotrichum*, *Penicillium, and Saccharomyces* with IFN-γ and IL-17 expression

Further analysis of the correlation between key microbiota and cell cytokines, we selected 6 genera with potential values for diagnosing TB to perform Linear Regression. The results show that *Bacteroides* presented a negative correlation with IFN-γ (*r*=−0.5958), and a positive correlation with IL-17 (*r* = 0.5997). *Blautia* and *Eubacterium_hallii_group* exhibited a positive correlation with IFN-γ (*r* = 0.4413, *r* = 0.4690) and a negative correlation with IL-17 (*r*=−0.2742, *r*=−0.3615) (Fig. 6a). The serum cell cytokines also show the alterations between the TB and the HC groups. Thus, it is also strongly associated with intestinal fungal biomarkers (Fig. 6b). *Apiotrichum* and *Penicillium* depicted a positive correlation with IFN-γ (*r* = 0.2840, *r* = 0.2618) and a negative correlation with IL-17 (*r*=−0.2369, *r*=−0.3538). *Saccharomyces* exhibited a negative correlation with IFN-γ (*r*=−0.3925) and a positive correlation with IL-17 (*r* = 0.4726). All the data generated indicate that gut microbial dysbiosis in TB patients may induce the alteration of IFN-γ and IL-17 in serum.

## Discussion

The intestinal microbiome is a crucial “organ” of the human that plays a vital role in the body’s health. However, most of the reported studies indicate that TB patients have gut microbial alteration, but the results of gut microbial dysbiosis in different research are not aligned and consistent with each other. Genetic characteristics and lifestyle may be the main reasons for these effects [20–22]. Therefore, it is indispensable to research the gut microbiota of TB patients in different regions.

The present study findings indicate that the diversity indexes of intestinal microbiota showed decreased levels in TB patients. The relative abundance of *Firmicutes* and *Bacteroidetes* decreased and increased respectively in TB patients, compared with the healthy subjects, which is consistent with the reported literature [16]. However, a cohort study of the Pakistani population showed that the abundance of *Firmicutes* was enriched, while reduced levels of *Bacteroidetes* in TB patients [18]. By comparing the two studies, we observed that the cohort study of the Pakistani TB population was accompanied by diabetes. Diabetes is known to be a major influencing factor in gut microbial dysbiosis [23]. Hence, it is assumed that the differences in gut microbial composition might relate to the disease factor of the study subjects.

Fungal flora contributes 0.1% of intestinal microbiota, which plays an important role in the maintenance of gut microbial ecology equilibrium [24]. Such literature is scarce that explains the relationship between intestinal fungi and TB. Current research results indicated that the diversity of intestinal fungal flora was significantly reduced, and gut microbial composition was also significantly altered in the TB group vs. healthy subjects. Our data revealed that the abundance of *Ascomycota* was dramatically increased, while *Basidiomycota* was lowered in TB patients. These results disagreed with other reported literature, where *Basidiomycota* abundance was significantly increased in 29 TB patient samples [25].

The interaction between bacteria and fungi affects intestinal homeostasis, which is vital in maintaining health [26]. In the current study, we focused on the collective gut bacterial microbiota and fungi mycobiota and also performed a cross-kingdom network analysis. The results showed that the ITS/16S ratio and the complexity of the trans-kingdom network significantly decreased in TB patients compared to healthy subjects. Moreover, through the ROC model prediction, we find that a combination set of three bacterial genera (*Bacteroides, Blautia*, and *Eubacterium_hallii_group*) and three fungal genera (*Apiotrichum, Penicillium*, and *Saccharomyces*) had remarkable efficacy to distinguish the TB patients from healthy humans (AUC = 0.996). Hence, it is more appropriate to study both gut bacteria and fungal microbiota as a whole for diagnostics biomarkers of TB patients.

Through the respiratory tract, *M.tb* invades the lungs and induces the immune response in the host dominated by the pro-inflammatory mechanism to relieve, isolate, and kill pathogens [27, 28]. IFN-γ is one of the common pro-inflammatory cytokines that play a key role in controlling the *M.tb* infection through mediating immune response resulting in the activation of macrophages [29]. Previous reported studies explain that IFN-γ level was significantly increased in the TB patient’s serum compared to the healthy group [30, 31]. It is worth noticing that IFN-γ levels appear to be negatively correlated with disease severity. TB patients co-infection with HIV had much lower IFN-γ levels than those having only TB infection alone [27]. Our findings also indicate that the IFN-γ level has significantly been reduced in the TB patient’s serum compared to the healthy people. In addition, another pro-inflammatory factor, IL-17, is also important for the formation of mature granulomas in *M.tb* infection [32]. In the present study, we noticed that the IL-17 levels in TB patients were higher than in the healthy subjects, which is accorded with previous literature [33]. Excessive expression of IL-17 can aggravate the inflammation, which enhances neutrophil recruitment and tissue damage [34]. The variations in the findings of cytokine-mediated immune responses in the development of TB need to be further studied in the future.

However, our study revealed a clear-cut alteration of gut microbial composition in TB patients. The specific mechanisms through which gut microbial dysbiosis affects immune functions are still unclear. Specific gut microbiota plays a vital role in inducing different immunophenotypes or cytokine responses, which may influence the pathogenesis or the pathology of the disease [35, 36]. It has been documented that gut microbial alteration can affect the host’s immune function and immunity against the disease [37, 38]. The present study illustrates the notable dysbiosis of the gut microbiome (including bacteria and fungi) in TB patients, which is also associated with serum IFN-γ and IL-17 levels. Enriched prevalence of *Bacteroides* in TB patients is negatively correlated with IFN-γ and positively correlated with IL-17. *Bacteroides* produce a higher quantity of SCFAs in the gut, which may enhance TB susceptibility by suppressing the B cells and CD4 + and CD8 + lymphocytes, ultimately reducing the production of TB-induced IFN-γ [12, 39, 40]. In addition, *Bacteroides fragilis* plays a significant role in the maturation of Th1-driven immune responses through increasing CD4 + T cell and IFN-γ production [11]. In the current study, we observed a raised level of *Blautia* in healthy controls. It is positively correlated with IFN-γ and negatively correlated with IL-17. It has been proven that *Blautia* has a strong ability to inhibit proinflammatory reactions (IL-17) by producing butyrate [41, 42]. After *M.tb* infection, it has been found that reduced relative abundance of *Prevotella* limits butyrate production. Hence, it may lead to a higher expression of IL-17 in TB patients. Moreover, *Prevotella* possessed the ability to regulate Th17 cell responses [43]. In *M.tb* infection, a higher abundance of *prevotella* may further induce Th17 cell response and promote the production of IL-17. When Th17 cells produce excessive IL-17, a large number of neutrophils are recruited and clustered together to create the tuberculosis pathological nodules [34]. Therefore, targeting to recover the immune cytokines by regulating the intestinal microbiome may have a potential strategy to assist the TB treatment. A further in-depth investigation of the gut microbiome is needed to explain the exact mechanism of diagnosis and treatment of TB patients.

## Conclusions

In conclusion, the present study reveals that (i) the intestinal bacterial and fungal dysbiosis was observed in TB patients. (ii) the equilibrium between gut bacteria and fungi was notably altered, and the interactions among bacterial-fungal trans-kingdom were also reduced in TB patients. So, gut microbiota should be studied as a whole to observe the function of gut microbiome. (iii) the genera including *Bacteroides, Blautia, Eubacterium_hallii_group, Apiotrichum, Penicillium*, and *Saccharomyces* depict the notable alterations in TB patients. A set containing these 6 genera has the potential ability to diagnose TB. (iv) Some dysbiotic gut microbes are strongly associated with the alteration of IL-17 and IFN-γ, which may be involved in the development of TB. Hence, regulating the intestinal microbial equilibrium may be a potential strategy for treating TB patients.

### Electronic supplementary material

Below is the link to the electronic supplementary material.

Supplementary Material 1

Supplementary Material 2

## Data Availability

Sequence data that support the findings of this study have been deposited in the Sequence Read Archive (SRA) of the National Center for Biotechnology Information (NCBI), the BioProject number PRJNA996946.
