# Supplementary material for: Gut bacterial and fungal dysbiosis in tuberculosis patients
Source: BMC Microbiol. 2024 Apr 25;24:141. doi: 10.1186/s12866-024-03275-8 (PMC11044546; doi:10.1186/s12866-024-03275-8)
Supplement: Supplementary file 1 — Supplementary Material 1 [file 12866_2024_3275_MOESM1_ESM.pdf]

Supplemental Tables and Figures

Table S2. The relative abundance of bacteria in TB and HC groups at phylum levels

phylum name	TB-mean(%)	HC-mean(%)	P_value
p__Firmicutes	58.11	66.24	0.155
p__Proteobacteria	9.398	19.11	0.1044
p__Bacteroidota	24.02	2.263	1.46E-06
p__Actinobacteriota	5.963	12	0.004326
p__Fusobacteriota	1.277	0.009737	0.06611
p__Synergistota	0.5824	0.002554	0.7768
p__Verrucomicrobiota	0.3275	0.1074	1
p__Desulfobacterota	0.2287	0.05699	0.1272
p__Patescibacteria	0.02128	0.136	3.84E-07

Comparisons of the relative abundances of intestinal bacteria between the TB and HC groups were conducted at the phylum levels (Wilcoxon Rank Sum test), TB: tuberculosis; HC: healthy control.

Table S3. The relative abundance of bacteria in genus level

genus name	TB-mean(%)	HC-mean(%)	P_value
g__Bacteroides	13.63	1.795	1.68E-05
g__Prevotella	8.035	0.01277	0.002179
g__Escherichia-Shigella	7.285	13.53	0.0895
g__Faecalibacterium	6.927	6.027	0.9487
g__Blautia	6.601	13.36	0.0001037
g__Megamonas	5.411	0.04837	0.9206
g__Bifidobacterium	4.742	7.959	0.01173
g__Veillonella	4.289	0.1772	0.1655
g__Subdoligranulum	3.796	6.597	0.01489
g__Phascolarctobacterium	2.437	0.0573	0.09631
g__Roseburia	2.333	0.8862	0.617
g__Lactobacillus	1.913	0.8072	0.432
g__Clostridium_innocuum_group	1.689	0.05794	4.00E-05
g__Dialister	1.543	0.1227	0.5056
g__Enterococcus	1.461	0.2123	0.6806
g__Lachnoclostridium	1.442	0.1518	0.00818
g__unclassified_f__Lachnospiraceae	1.302	0.728	0.4517
g__Fusobacterium	1.277	0.009418	0.0658
g__Agathobacter	1.144	2.397	0.002732
g__Erysipelatoclostridium	1.116	0.0083	0.0001058
g__Klebsiella	0.9926	3.815	0.001527
g__Eubacterium_hallii_group	0.9166	4.313	1.46E-05
g__Ruminococcus_gnavus_group	0.8855	1.047	0.7867
g__Ruminococcus_torques_group	0.8817	1.598	0.001862

g__Anaerostipes	0.8808	1.555	0.0006838
g__Collinsella	0.8325	2.999	0.001058
g__Alistipes	0.6959	0.2602	0.3482
g__Streptococcus	0.6755	1.247	0.006078
g__Butyricicoccus	0.6575	0.5232	0.1055
g__Ruminococcus	0.6328	2.692	0.00306
g__Megasphaera	0.5929	0.9507	0.9148
g__Dorea	0.5844	3.987	9.54E-06
g__Lachnospiraceae_NK4A136_group	0.5838	0.3236	0.3179
g__Pyramidobacter	0.5809	0.0006385	0.8546
g__Fusicatenibacter	0.5606	3.142	4.61E-05
g__Parabacteroides	0.5377	0.08252	0.0007242
g__norank_f__Eubacterium_coprostanoligenes_group	0.5005	0.4956	0.1674
g__Parasutterella	0.4935	0.08492	0.008549
g__Haemophilus	0.482	0.04326	0.8057
g__Romboutsia	0.4805	3.435	5.00E-05
g__Coprococcus	0.3861	0.7058	0.009789
g__Akkermansia	0.3269	0.1074	0.791
g__Paraprevotella	0.302	0.01277	0.6974
g__Catenibacterium	0.2922	0	0.1765
g__Prevotellaceae_UCG-003	0.2484	0	0.2785
g__Intestinibacter	0.248	0.8592	0.03976
g__UCG-002	0.2317	0.1137	0.992
g__Staphylococcus	0.2316	0	0.07331
g__Barnesiella	0.2313	0.05507	0.6596
g__Eubacterium_ventriosum_group	0.2225	0.1253	0.961
g__norank_f__Ruminococcaceae	0.2211	0.06481	0.774
g__Eubacterium_eligens_group	0.2188	0.019	0.4837
g__Erysipelotrichaceae_UCG-003	0.2008	1.421	2.47E-06
g__Flavonifractor	0.199	0.01101	0.0004114
g__Clostridium_sensu_stricto_1	0.1949	2.154	0.000218
g__Bilophila	0.1794	0.007502	0.01339
g__Eubacterium_siraeum_group	0.1565	0.01038	0.2639
g__Clostridioides	0.1499	0	0.04719
g__Adlercreutzia	0.1469	0.5995	0.01332
g__NK4A214_group	0.1396	0.1318	0.1499
g__Christensenellaceae_R-7_group	0.135	0.01341	0.0875
g__Holdemanella	0.1348	0	0.04721
g__Monoglobus	0.1293	0.6722	0.003405
g__Terrisporobacter	0.119	0.1705	0.07577
g__UCG-005	0.1163	0.006545	0.2069
g__Ruminococcus_gauvreauii_group	0.1141	0.4013	0.0001447
g__CAG-56	0.1108	0.3692	0.02554
g__Sellimonas	0.108	0.005587	0.05243

g__Oscillibacter	0.1021	0.004469	0.02156
g__Turicibacter	0.09761	0.3269	0.01344
g__unclassified_f__Enterobacteriaceae	0.07778	1.001	0.01201
g__norank_f__Coriobacteriales_Incertae_Sedis	0.03009	0.1338	0.181
g__Eisenbergiella	0.02128	0.1612	0.9413
g__CAG-352	0.01877	0.363	0.3026
g__Enterobacter	0.003773	0.6233	1.57E-05
g__Weissella	0.002225	0.1528	8.81E-08
g__Pediococcus	0	0.103	0.07089

Comparisons of the relative abundances of intestinal bacteria between the TB and HC groups were conducted at genus levels (Wilcoxon Rank Sum test), TB: tuberculosis; HC: healthy control.

Table S4. The relative abundance of fungi at the phylum level

phylum name	TB-mean(%)	HC-mean(%)	P_value
p__Ascomycota	84.89	68.46	0.000781
p__unclassified_k__Fungi	11.18	17.88	0.008158
p__Basidiomycota	3.71	12.33	0.000206
p__Mucoromycota	0.2235	1.187	0.0103
p__Chytridiomycota	0.0001815	0.1358	0.01564

Comparisons of the relative abundances of intestinal fungus between the TB and HC groups were conducted at the phylum levels (Wilcoxon Rank Sum test), TB: tuberculosis; HC: healthy control.

Table S5. The relative abundance of fungi at the genus level

genus name	TB-mean(%)	HC-mean(%)	P_value
g__Saccharomyces	53.67	14.28	0.002249
g__Candida	21.61	22.26	0.432
g__unclassified_k__Fungi	11.18	17.88	0.008158
g__Pichia	3.249	0.7105	0.4941
g__Aspergillus	1.884	7.49	0.01224
g__Cutaneotrichosporon	1.64	4.64	0.01951
g__unclassified_p__Ascomycota	1.316	9.142	0.004802
g__Apiotrichum	0.9514	5.095	0.002257
g__Talaromyces	0.5429	0.02751	0.5779
g__Fusicolla	0.482	0.001495	0.768
g__Rhodotorula	0.2562	0.2612	0.008303
g__Starmerella	0.2446	0.0001495	0.768
g__unclassified_f__Trichosporonaceae	0.2339	0.0001495	0.5534
g__Penicillium	0.2057	1.604	0.000562

g__Eremothecium	0.1755	0	0.4633
g__Trichosporon	0.1648	0.08178	0.05354
g__Cladosporium	0.1594	0.5631	0.008389
g__Trichoderma	0.1509	2.055	0.1176
g__unclassified_o__Saccharomycetales	0.1465	0.205	0.4502
g__Alternaria	0.1285	1.783	0.03333
g__Vanrija	0.1159	0.02033	0.3791
g__Diutina	0.1005	0.01435	0.8923
g__Malassezia	0.04557	0.4294	0.001765
g__Dipodascus	0.04303	4.058	0.254
g__unclassified_o__Malasseziales	0.0413	0.1567	0.7184
g__Mucor	0.0335	0.1247	0.04872
g__Rhizopus	0.02805	0.6758	0.009821
g__Coprinellus	0.02079	0.2779	0.03947
g__unclassified_f__Didymellaceae	0.01707	0.3195	0.001223
g__Xeromyces	0.01371	0.3736	0.006748
g__Wallemia	0.01307	0.1529	0.352
g__unclassified_o__Chaetothyriales	0.01262	0.3259	0.4167
g__unclassified_f__Dipodascaceae	0.009985	0.5444	0.1001
g__unclassified_f__Aureobasidiaceae	0.00826	0.05083	0.6186
g__unclassified_f__Aspergillaceae	0.007444	0.302	0.03478
g__Exophiala	0.005174	0.1867	0.1122
g__Auricularia	0.002632	0.1944	0.274
g__unclassified_p__Chytridiomycota	0.0001815	0.1355	0.04374
g__Periconia	0	0.3487	0.2159
g__Gilbertella	0	0.3084	0.2159
g__Curvularia	0	0.2157	0.2159
g__Flammulina	0	0.1843	0.2159
g__Filobasidium	0	0.1745	0.008496
g__Xerochrysium	0	0.1601	0.000939
g__Lodderomyces	0	0.1334	0.008496

Comparisons of the relative abundances of intestinal fungus between the TB and HC groups were conducted at the genus levels (Wilcoxon Rank Sum test), TB: tuberculosis; HC: healthy control.

Figure S1. The different bacterial functions were evaluated between the two groups (Welch's t-test, p<0.05).

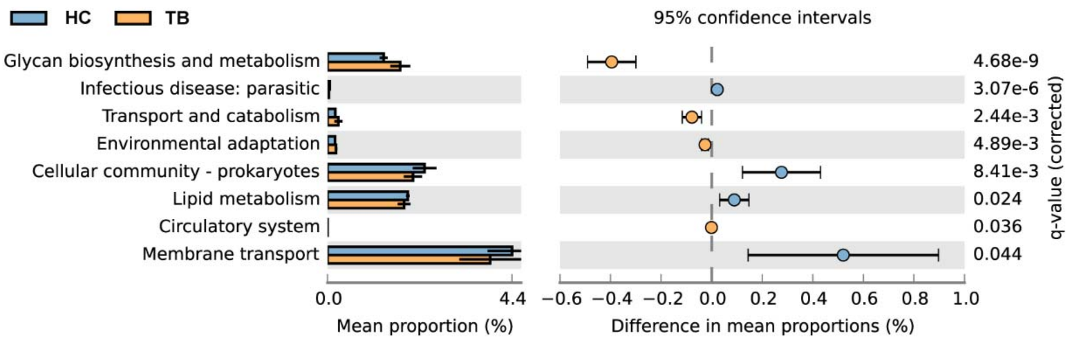

The potential enriched pathways of the microbiota were predicted using PICRUSt2 based on the 16S rRNA gene. The different bacterial functions were evaluated between the two groups with Welch's t-test. TB: tuberculosis; HC: healthy control.

Figure S2. The different fungal functions were evaluated between the two groups (Welch's t-test, p<0.05).

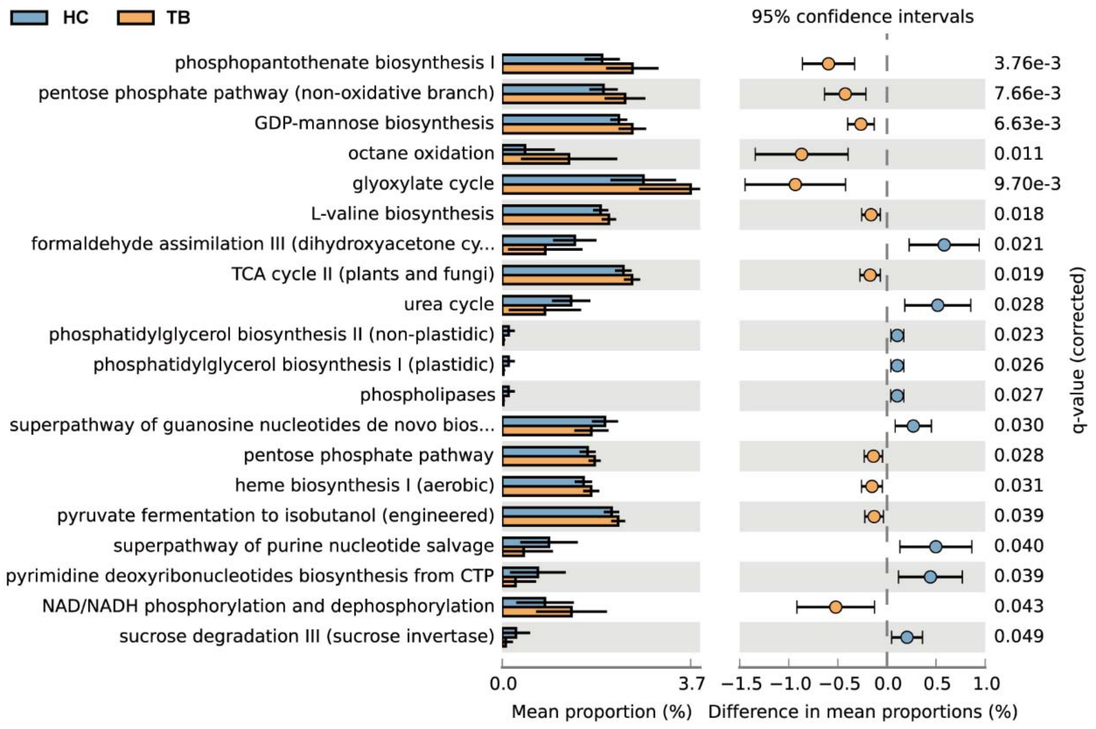

The potential enriched pathways of the mycobiota were predicted using PICRUSt2 based on the ITS2 gene. The different bacterial functions were evaluated between the two groups with Welch's t-test. TB: tuberculosis; HC: healthy control.
